# THE SOCIAL DETERMINANTS OF LATE-LIFE MIGRATION IN THE U.S.: FINDINGS FROM THE NEW AMERICANS PROJECT

**DOI:** 10.1093/geroni/igz038.1904

**Published:** 2019-11-08

**Authors:** Arati Maleku, Megan Espana, Sharvari Karandikar, Njeri Kagotho, Rupal Parekh, Shannon Jarrott

**Affiliations:** 1 The Ohio State University, Columbus, Ohio, United States; 2 University of Connecticut, Hartford, Connecticut, United States

## Abstract

Globally, late-life migration has been a growing phenomenon. Literature on aging and migration however, has primarily focused on immigrant populations who migrated early in life. To expand our conceptualization of aging and to plan for the care of growing older immigrant populations, it is crucial to understand the compounding effects of late-life migration and aging in new spaces. Drawing on the qualitative data (N=71) from a large-scale community-based participatory research project in a mid-western U.S. region, we examined the social determinants of late-life migration on the health and well-being of older immigrants by exploring: (a) barriers and facilitators of socio-cultural adaptation, (b) patterns of human service provision in a local context, and (c) societal patterns of caring for older immigrants in places of relocation. Life course and social convoy perspectives formed the conceptual basis of the study. Using Respondent Driven Sampling method, data collection included six focus group discussions (n=48) with immigrant communities and in-depth interviews with human service providers (n=23). Data analysis followed the Rapid and Rigorous Qualitative Data Analysis technique that generated six salient themes: cultural context of aging; challenges of late-life migration; broken convoy and social isolation; gender and age intersections; human services, and community efforts and solutions. Findings suggest that late-life migration is a conglomeration of losses and gains, contingent on complex determinants such as living arrangements, language, transportation, the built environment, inter-generational relationships, socio-economic status, and social convoys. We conclude with a call to develop age-friendly, culturally responsive human services and health policies.

